# The contribution of legal medicine in clinical risk management

**DOI:** 10.1186/s12913-018-3846-7

**Published:** 2019-02-01

**Authors:** Matteo Bolcato, Giacomo Fassina, Daniele Rodriguez, Marianna Russo, Anna Aprile

**Affiliations:** Department of Molecular Medicine, Legal Medicine, University of Padua, Padua, Italy

**Keywords:** Clinical risk management, Sentinel event, Adverse event, Professional liability, Medico-legal evaluation

## Abstract

**Background:**

In advanced health services, a main objective is to promote the culture of safety and clinical risk management. In this regard, the reporting of sentinel events fits within a perspective of error analysis, attempting to propose solutions aimed at preventing a new occurrence of the harmful event. The purpose of this study is to analyze the contribution of medico-legal litigation in the management of clinical risk and to propose an organizational model so as to coordinate the intervention of clinical risk management and medico-legal services.

**Methods:**

Retrospective review of 206 cases of medico-legal litigation, settled against a Hospital of a North-eastern city in Italy from January 1, 2014 and December 31, 2015.

**Results:**

Approximately 20% of cases, that are classifiable as “sentinel events”, were not reported due to various factors. The reason that these events are under-reported is mainly due to the latency between the event itself and its manifestation as a serious damage to health as well as the discomfort in reporting the events of this kind, which is still widespread among healthcare workers.

The systematic research of the available documentation for medico-legal purposes permits the acquisition of more information concerning the clinical event, thereby increasing the number and accuracy of the reports to the clinical risk unit.

**Conclusion:**

The analysis of medico-legal litigation is a valid tool to enhance the reporting of “sentinel events”. One possible proposal is the implementation of an organizational model to establish a rapid procedure for the reporting of sentinel events during the evaluation of medico-legal litigations.

## Background

In countries with advanced economic systems, the strategies aiming at the renovation and evolution of healthcare services are based on organization and planning.

Clinical risk management (CRM) is an organizational response plan aimed at improving the quality and safety of healthcare services by identifying the circumstances that put patients at risk of harm and then acting to prevent or control those risks. The CRM may be depicted as a 4-step process, where the risk is sequentially identified (1), analyzed in its severity and frequency (2), treated by reduction or elimination (3) and managed through the assessment of the costs both saved and raised by the reduction and, respectively, the occurrence of the risk (4) [1].

A key strategy of CRM is the building of a reporting system aimed at collecting and monitoring adverse event information and, in particular, sentinel events (SE), which are defined as “any unanticipated event in a healthcare setting resulting in death or serious physical or psychological injury, or the risk thereof … not related to the natural course of the patient’s illness or underlying condition” [2].

An enhancement of this clue strategy may consist in the close cooperation between CRM and legal medicine (LM) services, whose common purpose is the delivery of high-quality healthcare to patients**.**

Among the various competences of LM **(**patient rights, ethics, research, quality assurance, risk management, malpractice)**,** the evaluation of professional liability cases and lawsuits may help to identify potentially high-risk areas of medical practice and to enrich information regarding adverse events. Such an interdependent relationship between CRM and LM may constitute an effective informative net with regard to the critical issue of the system [3].

The Italian National Health System (NHS) delegates part of CRM functions to the New Health Information System [Nuovo Sistema Informativo Sanitario (NSIS)] that monitors the occurrence of SE and malpractice claims, developing, as a secondary objective, healthcare guidelines and specific measures to prevent or minimize the risks [4]. Table 1 reports the list of SE, according to a document drawn up by the Italian Ministry of Health [5]. The same document specifies that SE are also the events that determine serious damage with outcomes or clinical conditions that drastically affect the healthcare assistance pathway. These specific conditions are reported in Table 2.

**Table 1:** List of sentinel events with related numeration

1. Procedure on the wrong patient 2. Surgical procedure on the wrong part of the body (side, organ or part) 3. Incorrect procedure on the correct patient 4. An instrument or other material left within the surgical site that requires subsequent surgery or further procedures 5. Transfusion reaction consequent to AB0 incompatibility 6. Death, coma or serious damage deriving from errors in pharmacological treatment 7. Maternal death or serious illness related to labor and / or delivery 8. Death or permanent disability in a healthy, > 2500 g weighted newborn, unrelated to congenital illness 9. Death or serious damage due to the patient falling 10. Suicide or attempted suicide of the patient in hospital 11. Violence on the patient 12. Acts of violence against the healthcare professionals 13. Death or serious damage consequent to the malfunctioning of the transport system (intra-hospital, outside the hospital) 14. Death or serious damage consequent to an incorrect triage code assigned by the 118 Operating Center and/or the Emergency Ward 15. Death or serious unexpected damage consequent to surgical intervention 16. Any other adverse event that causes death or serious damage to the patient

**Table 2:** Outcomes or clinical conditions determined by sentinel events with consequences in the healthcare assistance

▪ Death ▪ Permanent disability ▪ Coma ▪ State of illness that determines prolonged hospitalization or chronicization ▪ Major trauma following the fall of a patient ▪ Transfer to a semi-intensive or intensive care unit ▪ Surgical reintervention ▪ Cardiorespiratory resuscitation ▪ Request for specific psychiatric and psychological treatments as a result of suicide attempts or violence suffered within the structure ▪ Transfusion reaction consequent to AB0 incompatibility ▪ Other unlisted changes (i.e. therapeutic treatments with additional drugs that would otherwise not have been necessary; request for diagnostic investigations of greater complexity; traumas and fractures)	

CRM and LM generally are generally distinguished as two units; rarely the two functions are embedded in a single structure. This division may result in an ineffective collection of information.

The presented study pursues the following objectives: i) to verify how LM may contribute to the knowledge of the clinical risk, identifying the SE emerging from the analysis of the litigation and not previously reported; ii) to ascertain the characteristics of the identified SE and understand the reasons for the previous failure of notification; iii) to propose an organizational model for a synergistic management of the litigations, involving both the ML and CRM units.

## Methods

This study, designed as a retrospective model, analyzed 206 compensation claims settled from 01.01.2014 to 31.12.2015 for alleged health professional liability against a hospital located in a northeastern Italian city, where two separate units manage CRM and LM functions. This casuistry was inserted into a computer database, named REDCAP (Research Electronic Data CAPture), which provides both an archive function, allowing an ordered collection and a rapid consultation of the inserted information, and a management function, permitting the reworking of the data for statistical analysis.

The information particularly concerned the clinical, administrative and judicial documentation, which included the charge advanced by the counterparty.

A medico-legal analysis was performed by comparing the individual clinical cases with the descriptive ministerial table of SE, in order to detect if situations ascribable to these latter occurred.

The verification of whether these situations had already been identified as SE was then carried out; furthermore, if the identification had been missed, a search for the reasons of the previous failure was also carried out.

## Results

The analysis of medico-legal litigation detected 16 SE: 9 had already been reported to CRM Unit, while 7 had not. These seven cases were notified to CRM professionals and confirmed as SE.

In the evaluated period (January 1, 2014 - December 31, 2015), further 19 SE were reported only to CRM Unit. They were related to events not included in the medico-legal litigation examined, such as attempted suicide occurring in hospital, assault of a health worker, etc. Therefore, the total number of SE amounted to 35.

Out of the total number, 28 SE had already been reported to CRM unit, while 7 had previously gone unreported: this means a 20% out of a total of 35 SE occurred in the considered period.

Out of the 16 SE that emerged from the present study, 11 pertain to the surgical area.

The other 5 are divided as follows: 2 pertaining to the emergency areas, one to the area of dentistry and 2 are non-healthcare-related. The latter category includes events that are related to infrastructure, transportation and organization of the environment.

In Table 3, we report the schematic description of the 16 SE emerged from the medico-legal analysis of the litigation. The 7 cases not registered in the CRM database (numbers 1, 5, 7, 10, 11, 12 and 15) are highlighted with a gray background.

**Table 3:**
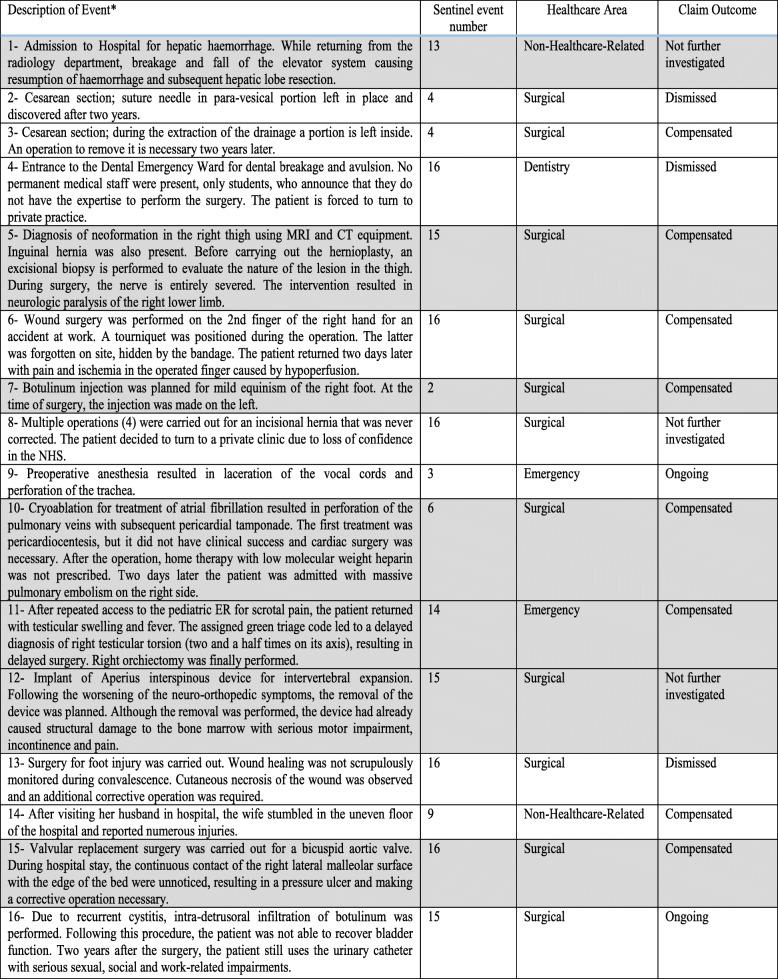
Brief description of sentinel events derived from the medico-legal study of the litigation

*All the descripted events occurred to patients aged between 20 and 90 years old, with a ratio of males to females 1:1

Concerning the outcome of the SE, 8 claims were compensated, 3 were dismissed, 2 were not further investigated and another 2 were still under assessment.

In the group of 7 SE identified only by ML Unit, 5 were accepted and 2 did not undergo further investigation.

## Discussion

The reporting systems of adverse events in the healthcare context are an essential tool for increasing knowledge of the causes and contributing factors pursuant to the principle that “[...] no one can avoid making mistakes; the best thing is to learn from them” [6, 7]. In this context, the monitoring of SE is aimed at collecting information regarding potentially avoidable adverse or near-adverse events of particular severity, which can result in death or serious harm to the patient and determine a citizens’ loss of confidence in the NHS, not only in Italy [8–11].

The analysis of medico-legal litigation proves to be an excellent tool, with high accuracy and reliability, useful for the detection of situations that have previously gone unrecognized and unrecorded as SE by CRM professionals.

In particular, referring to point i) of the aims of this study, it can be stated that the analysis of medico-legal litigation was characterized by an excellent accuracy; in fact, among the SE that had given rise to a claim of medical liability and had been previously reported, no case escaped our attention.

Furthermore, our research helped to bring to the attention of CRM some previously missed cases. It is imperative to underline that 5 cases out of 7 were compensated, suggesting a fundamental role of ML unit in recognizing SE with a high-profile impact to both the hospital and the patient.

Out the 16 cases reported in the presented study, 2 have given rise to the following substantial improvements in daily clinical practice: 1) changes in protocols of drainage removal in Obstetrics Department, such as the mandatory comparison between the length and number of draining windows of the extracted drainage with the data of the operating room (Case No. 3); 2) and the extension of the use of a dermographic pencil in the marking procedure of the surgical site to the percutaneous injections of botulinum toxin (Case No. 7).

Not least, it should be noted that SE are primarily focused in the surgical area. 11 out of the 16 cases (69%) emerged from the analysis of medico-legal litigation belong to this area, confirming it as a high-risk area in the healthcare framework. The events are evenly distributed among the various categories of SE. Indeed, there is no type of SE repeated such a number of times as to raise the hypothesis that serious critical issues are concentrated in a single activity within the hospital context.

Concerning aim ii), the reasons for failure to report the SE, before the medico-legal analysis was carried out, are multiple. One reason is connected to the definition of serious damage as a prerequisite to qualify the event as “sentinel”. Nevertheless, the damage may be manifest in all its severity after several days or even years after the critical event: its real significance may therefore not be initially understood.

From Table 3, it is evident that in 5 (numbers 1,7,10,11 and 15) out of the 7 cases qualified as SE and initially unreported, there was no serious damage in the early stages of the clinical episode. It is not uncommon for the sequelae of a specific event to become evident only at considerable distance from the fact itself. When this occurs, a claim very often intervenes, permitting an awareness of the consequences of a specific event even after many years.

An additional motivation lies in the fact that, not infrequently, the event that could have the features of SE is not reported to CRM by the health professionals involved, as they fear that such a report could determine the identification and blame of those professionals who are responsible, as in cases 5 and 11. This motivation should not exist, since the actions of CRM are not promoted in order to blame health professionals, but to study strategies in order to avoid the recurrence of the error. However, the analysis of such litigation is not able, independently, to identify all SE occurring within the hospital setting, since not all SE give rise to requests for compensation. Thus, reporting by healthcare professionals remains an essential informational tool for the proper functioning of the system [12–16]. In order to improve the implementation of prevention strategies by healthcare companies it is necessary to continue to promote the culture of CRM in healthcare professionals.

Thanks to the interventions of CRM it has been possible to develop protocols and procedures targeted at specific situations, with the aim of reducing the incidence of certain errors. The greater the number of cases being analyzed, the greater the knowledge regarding error prevention, and thus the more effective the related solutions.

The experience described refers to the social organization in which we operate and which sees the units of CRM and LM as structurally separate. Such a separation does not exist in many Italian hospital structures, since the complementarity of these activities has been recognized [17, 18].

Regardless of the opportunity to bring these activities into a single structure, it is important to ensure, in all hospital centers, an organizational system aimed not only at implementing cooperation and data exchange for the achievement of an adequate service of CRM, but above all to create a new *modus operandi*, integrating the various aspects that emerged with different approaches [19].

## Conclusion

The analysis of medico-legal litigation proves to be an excellent tool, with high accuracy and reliability, useful for the detection of situations that have previously gone unrecorded and unrecognized as SE by CRM professionals.

The medico-legal approach, through its peculiar analytical method, has drawn attention to high profile SE previously undetected with the customary tools of CRM.

We believe it would be opportune to elaborate protocols that (1) describe the data – obtaining them from the constitutive parameters of the various SE - to be collected in individual cases, also specifying the time in which they should be collected, and (2) specify the methodology of their analysis, in two different activities: clinical risk management in the strict sense and medico-legal litigation.

Our research has shown that the medico-legal expert – however much restricted to those cases which have given rise to a dispute - is privileged to be able to make use of an analysis of cases as they evolve over time.

## Abbreviations

CRM: Clinical Risk Management
LM: Legal Medicine
NHS: National Health Service
NSIS: Nuovo Sistema Informativo Sanitario (New Health Information System)
REDCAP: Research Electronic Data CAPture
SE: Sentinel Events
